# Three-Dimensional iPSC-Based In Vitro Cardiac Models for Biomedical and Pharmaceutical Research Applications

**DOI:** 10.3390/ijms251910690

**Published:** 2024-10-04

**Authors:** Simona Bufi, Rosaria Santoro

**Affiliations:** 1 Unit of Vascular Biology and Regenerative Medicine, Centro Cardiologico Monzino-IRCCS, 20138 Milan, Italy; 2 Department of Electronics, Information and Biomedical Engineering, Politecnico di Milano, 20133 Milan, Italy

**Keywords:** in vitro modeling, cardiac, organoid, microtissue, engineered heart tissue, cardiomyopathy

## Abstract

Cardiovascular diseases are a major cause of death worldwide. Advanced in vitro models can be the key stone for a better understanding of the mechanisms at the basis of the different pathologies, supporting the development of novel therapeutic protocols. In particular, the implementation of induced pluripotent stem cell (iPSC) technology allows for the generation of a patient-specific pluripotent cell line that is able to differentiate in several organ-specific cell subsets while retaining the patient genetic background, thus putting the basis for personalized in vitro modeling toward personalized medicine. The design of iPSC-based models able to recapitulate the complexity of the cardiac environment is a critical goal. Here, we review some of the published efforts to exploit three dimensional (3D) iPSC-based methods to recapitulate the relevant cardiomyopathies, including genetically and non-genetically determined cardiomyopathies and cardiotoxicity studies. Finally, we discuss the actual method limitations and the future perspectives in the field.

## 1. Introduction

Despite the considerable steps forward in the field, cardiovascular diseases are still a major cause of death and disability worldwide, with a strong impact on healthcare costs [1]. For this reason, the decryption of the mechanisms at the basis of cardiac pathologies and the identification of novel treatments are still hot research topics. Research sustainability is tightly linked to the availability of models successfully recapitulating pathological and physiological scenarios. Indeed, research and development allocations are still highly affected by the costs of compound discovery, preclinical studies, and clinical trials [2]. In particular, preclinical trials rely on the use of animal models, which are linked to high costs (e.g., for housing and handling), complicated management, and ethical concerns (the 3R principles of replacement, reduction, and refinement call for their limitation) and, due to interspecies variability, are often unable to successfully recapitulate some relevant aspects of human cardiac pathophysiology [3].

Advanced in vitro models could be an alternative to overcome some of these limitations. In particular, the implementation of induced pluripotent stem cells (iPSCs) provides several relevant advantages. iPSCs provide human cells, obtained from somatic, fully differentiated cells, collected from peripheral districts. Being pluripotent, they have the potential to differentiate in several cell subsets, thus allowing for the recapitulation of complex tissues and organs. Moreover, iPSCs carry the donor genetic content, and the joint use of iPSC and CRISPR/Cas9 technology, simplifying cell line genetic corrections, enables the design of complex experimental setups to investigate the effects induced by specific genetic defects.

The full exploitation of the great potential of these technologies is secondary to the ability to design in vitro models, allowing for the recapitulation of relevant organ features, such as complex geometry, multicellularity, intercellular interaction, and organ functionality, thus making the in vitro model representative of physiological and pathological human cardiac scenarios. Indeed, numerically, cardiomyocytes are only 20–30% of cells in the myocardium, where endothelial cells (64%), cardiac fibroblasts (27%), and leukocytes (9%) play a relevant role in cardiac structure and homeostasis [4]. The interaction between the different resident cardiac cells is guided by multiple factors, such as cell–cell junctions, cell–ECM interactions, released soluble factors, and geometrical and mechanical cues [5,6,7,8].

The use of traditional 2D static cultures of iPSC-derived single-cell types (e.g., cardiomyocytes), despite demonstrating their relevance for modeling the activation of biological mechanisms in response to stimuli, has been limited by the low level of maturity of iPSC-derived cardiomyocytes, which are mainly representative of an early developmental stage [9]. iPSC-derived cell maturity can be enhanced by geometrical cues, interaction with other cardiac cell types, and physical stimuli [10,11].

With the final goal to better mimic the cardiac tissue morphological, biochemical, and mechanical features to improve maturity, several 3D culture methods have been designed [12,13,14], based on the assembly of either primary cells or iPSC-derived cardiac subsets obtained by previous differentiation in 2D. Cells are assembled either in spheroids or microtissues, with or without the use of biomaterials (e.g., Matrigel and collagen); deposited through bioprinting methods or assembled in microwells; and cultured statically, using microfluidic chambers, or exposed to mechanical training (e.g., engineered heart tissues) [15,16]. Based on the experience in other fields (e.g., neurology, gastroenterology, and cancer) [17,18], in recent years, an increasing interest has risen in the design of methods for the production of cardiac organoids. By definition, organoids are 3D constructs typically derived from stem cells that take advantage of their innate ability to differentiate and self-organize following special restricted lineage commitment specifications. Organoids recapitulate in vivo tissue-like cellular heterogeneity and interconnection, tissue structure, and functionality, making them suitable for a wide variety of applications, ranging from drug discovery to personalized medicine. Despite their high potential, examples of organoids in the cardiac field are still limited, and only recently did they gain popularity.

**Figure 1:**
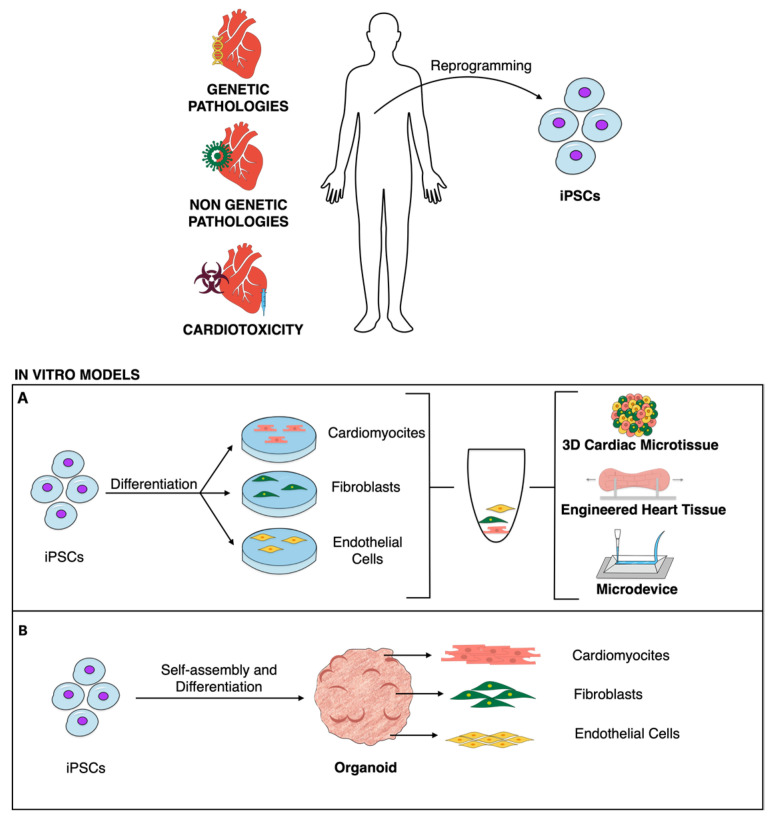
iPSC potential for in vitro modeling of cardiomyopathies can be exploited thanks to 3D models. These can be obtained (**A**) through assembly of cardiac cell subsets, previously differentiated in 2D, or (**B**) through self-assembly approaches.

In the following paragraphs, we will review the literature providing examples of the exploitation of organoids (ORGs), microtissues (MTs), and engineered heart tissues (EHTs) for in vitro modeling of some cardiomyopathies (Figure 1). Cardiomyopathies are a subgroup of cardiac diseases defined by structural or functional disorders of the myocardium in the absence of secondary causes of heart failure (e.g., hypertension and valvular diseases), and they can have a genetic or non-genetic origin [19]. We will provide an overview of the reported application of organoids for the decryption of the mechanisms at the basis of cardiomyopathies and for the identification of novel treatments. Moreover, we will discuss their potential and their limitations as preclinical screening platforms of novel drugs and for the design of personalized medicine protocols.

## 2. In Vitro Modeling Genetic Cardiomyopathies

iPSC technology enables the generation of cell lines carrying the patient genotype that, coupled with corrected gene-edited lines [20], make them the natural choice for modeling genetic disorders. Recently, the optimization of CRISPR-Cas9-based gene-editing methods, especially those based on new nucleases, which drastically reduce off-target effects, made the perfect control available, characterized by correction specificity, modifying only a known causative mutation or a putative genetic pathogenic mutation in the iPSC line. Several attempts to implement this potential by designing reliable in vitro models of genetic cardiomyopathies were reported (Table 1).

The most common genetic cardiomyopathies include hypertrophic cardiomyopathy (HCM; MIM e.g., #192600, 613765, 600858, and 115150), dilated cardiomyopathy (DCM; MIM e.g., #115200 and 604145), restrictive cardiomyopathy (RCM; MIM e.g., #115210), arrhythmogenic cardiomyopathy (ACM; MIM e.g., #609040 and 607450), non-compaction cardiomyopathy (NC; MIM e.g., #604169), and some inherited syndromes associated with cardiac disease (e.g., muscular dystrophy, MIM #310200 and 300375) [19].

HCM is a relatively common genetic cardiomyopathy, with an estimated prevalence of 1 in 500 people. It is characterized by left ventricular hypertrophy unexplained by secondary causes and a non-dilated left ventricle with a preserved or an increased ejection fraction [21]. The genes more commonly involved in variants causing familial HCM are *MYH*7, *MYBPC*3, *TNNT*2, and *TNNI*3.

A study by Buono et al. [22] reported the application of 3D MTs for HCM in vitro recapitulation. Two iPSC lines were obtained, respectively, from an HCM patient carrying a mutation in the gene encoding for myosin heavy chain 7 (*MHY*7) and from a healthy donor. Cardiomyocytes derived either from a patient and control iPSC line (iPSC-CM) were assembled in MT in a co-culture with primary human cardiac fibroblasts (CFs) and primary human cardiac microvascular endothelial cells (ECs) in a ratio resembling that of native adult cardiac tissue. MTs obtained using patient-derived iPSC-CMs showed a lower level of structural organization and presented an arrhythmic phenotype. The proposed method, despite its relevance, fails in the recapitulation and quantification of the structural modifications in the myocardium and of the related functional effects, such as the generated force.

An alternative approach to overcome these limitations is described by Cashman et al. [23], who proposed the use of iPSC-CMs carrying an HCM causative mutation for the generation of EHT. A comparison of the EHT obtained from healthy and patient-derived iPSCs showed several structural, molecular, and functional indications of a hypertrophic phenotype, such as significant differences in twitch dynamics and in diastolic force. The method has a potential for in vitro modeling and a personalized medicine approach; nevertheless, it has to be noted that some pathology descriptive features tend to diminish over the culture period. This limitation is common in iPSC-based methods due to the limited capacity of iPSC-CMs to reproduce a mature adult phenotype.

Using another MT model, Hinson et al. [24] modeled the role of a variant in *PRKAG*2 in the regulation of AMP-activated protein kinase (AMPK), leading to left ventricular hypertrophy, glycogen accumulation, and ventricular pre-excitation.

A more interesting approach to model this pathology was recently proposed by Meier et al. [25]. The authors designed a protocol for epicardioids, organoids constituted by epicardium and myocardium components, and they characterized the model for the recapitulation of both cardiogenesis (e.g., epicardium-related developmental processes) and for the in vitro modeling of HCM. Indeed, epicardioids could recapitulate fibrotic and arrhythmic phenotypes typical of HCM either in response to pharmacological stress or when iPSCs reprogrammed from a Noonan syndrome-affected patient were used.

DCM also is a common disease of the heart muscle; it is often classified as idiopathic and without an identifiable cause, although a familiar and genetic predisposition is demonstrated, and DCM has been associated with variants in genes encoding for Titin (e.g., *TTN*) [26], Desmin (e.g., *DES*) [27], Lamin (e.g., *LMNA*) [28], and Myosin (e.g., *MYH*7 and *MYH*6) [29,30].

Camman et al. [31] reviewed in detail the literature on the attempts to in vitro model the complexity of this pathology, underlying the importance of supporting cellular 3D tissue geometrical features (e.g., cell alignment), extracellular composition (e.g., collagen), and mechanical properties (e.g., stiffness). In this direction, the implementation of scaffold-based approaches was proposed, taking advantage, for example, of electrospun-based methods to deposit fibers of controlled dimensions, mechanical properties, and alignment. Indeed, the incorporation of all those features increases the degree of complexity of the described methods. An MT-based approach was proposed by Hinson et al. [32], who were able to recapitulate sarcomere insufficiency, resulting in altered basal contractility and increased adrenergic and contractile stress.

ACM is a rare syndrome (prevalence approximately 1 in 5000) associated with genetic abnormalities [33] mainly in the genes encoding for desmosomal proteins, including *PKP2* [34], *DSP* [35], *DSC2* [36,37], *DSG2* [38], and *JUP* [39]. Desmosomes are part of the intercalated disks, which are structures that guarantee mechanical and electrical coupling between adjacent cardiomyocytes and, finally, coordinated contraction of the whole heart [40]. Desmosomal genes are also expressed cardiac mesenchymal stromal cells, and their mutations have been correlated with adipose substitution [41]. Due to the dysregulation of these mechanisms, ACM is a progressive myocardial disease, characterized by fibro-fatty substitution, often resulting in an arrhythmic phenotype and leading to heart failure and sudden death. The diagnostic criteria of ACM still need to be refined and the mechanisms at the basis of this pathology should be better described in order to identify novel pharmaceutical targets and etiological treatments.

Giacomelli et al. [42] validated their method for the production of MTs by showing its applicability to recapitulate the role of non-cardiomyocytes in ACM. The MT is obtained by 3D assembly of tree cardiac cell subsets (CMs, cardiac fibroblasts, and endothelial cells), previously differentiated in 2D from iPSCs. This method allowed the authors not only to support iPSC-CM maturation in vitro but also to recapitulate the ability of cardiac fibroblasts carrying a desmosomal mutation, leading toward the development of an arrhythmic phenotype.

A major player in ACM is the mechanical stress; indeed, the pathological phenotype is often exacerbated by intense physical activity, showing the intrinsic fragility due to the desmosome inefficacy of the complex cinematic chain involved in heart contraction. In vitro recapitulation of the role of mechanical stress in ACM is not trivial. Bliley et al. [43] designed an EHT model enabling the regulation of pre-load and, using ACM patient-derived iPSC-CMs, showed its enhanced capacity to recapitulate stress-induced pathological remodeling.

Interest has risen in the in vitro modeling of cardiomyopathies secondary to inherited diseases, in particular genetic neuromuscular diseases, such as muscular dystrophy (MD). The impairment in dystrophin functionality leads to the development of a fibrotic and arrhythmic phenotype. These characteristics were reproduced in vitro by means of MT and EHT models.

Marini et al. [44] produced cardiac organoids from DMD-iPSCs: iPSC-spheroids were cultured in suspension following a cardiac differentiation protocol and a long-term culture. Through the culture period, the resident cells modified their phenotype, retracing some features of the pathogenesis of this cardiomyopathy, such as cell death, CM deterioration (e.g., calcium handling-related machinery impairment), and fibro-adipose tissue deposition. Moreover, the authors claim that their system shows the presence of a dystrophin-associated protein complex and other late cardiac differentiation markers, often missing in widely used EHT models. Nevertheless, EHT models have been successfully used to verify improvement in contractile functionality in response to gene editing to restore dystrophin secretion [45,46] or to verify mechanisms regulating DMD cardiomyopathy [47].

**Table 1:** Summary of main studies proposing advanced in vitro modeling of genetic cardiomyopathies.

Genetic Pathology	Genotype	Technique	Cell Subsets	Ref.
HCM	*MYH*7	MT	iPSC-CM EC CF	[22]
HCM	*BRAF*	EHT	iPSC-CM SC	[23]
HCM	*PRKAG*2	MT	iPSC-CM	[24]
HCM	*TTN*	CO	iPSC	[25]
DCM	*TTN*	MT	iPS-CM SC	[32]
ACM	*PKP*2	MT	iPSC-CM iPSC-EC iPSC-CF	[42]
ACM	*DSP*	EHT	iPSC-CM	[43]
DMD	*DMD*	CO	iPSC	[44]
DMD	*DMD*	EHT	iPSC-CM CF	[45]
DMD	*DMD*	EHT	iPSC-CM	[46]
DMD	*DMD*	EHT	iPSC-CM	[47]

## 3. In Vitro Modeling Non-Genetic Cardiomyopathies

Even in the absence of genetic variants, iPSCs can be crucial for the optimization of in vitro modeling of cardiomyopathies thanks to their ability to recapitulate the complexity of the cardiac tissue (Table 2).

Among the most common non-genetically determined cardiomyopathies is ischemic cardiomyopathy secondary to myocardium infarction (MI). MI due to reduced myocardial blood flow causes an irreversible loss of viable cardiac mass, resulting in a ventricular dysfunction with a prevalence of 10% in 2 years [48]. Several attempts were carried out to model this condition.

Richards et al. [49] used their previously described organoid model [50] as a post-MI model by modulating oxygen diffusion gradients and inducing chronic adrenergic stimulation. In response to these stimuli, a meta-analysis demonstrated a switch toward a post-MI phenotype, accompanied by coherent modifications in the metabolism, calcium handling, and endogenous fibrosis.

Similarly, the work of P. Sharma et al. [51] proposed a hanging drop protocol for the generation of MTs composed of iPSC-CMs, human CFs, and human coronary artery ECs. In this model, ischemia/reperfusion was simulated using pathophysiological oxygen concentrations (from 5% to 0% environmental oxygen concentration) and administering a toxic compound (Doxorubicin), successfully recapitulating some of the relevant pathological features, including cell death, impaired contractility, and modifications in the transcriptomic signature.

In vitro modeling of the mechanisms underlying the adulthood pathophysiology is complicated by the difficulties in supporting full maturation of iPSC-derived cells. In this regard, the work of Voges et al. [52] is interesting, as they proposed the use of EHT for the study of the mechanisms regulating the fetal cardiac tissue self-regenerative potential. In this model, in response to cryoinjury the activation of cardiomyocyte hypertrophy to restore the damage, without deposition of fibrotic tissue, can be observed.

Other common non-genetically determined cardiomyopathies are secondary to metabolic disorders and infection-induced cardiomyopathies. In the literature, there are some examples of the implementation of cardiac organoids for these applications.

For example, Lewis-Israeli et al. [53] used cardiac organoids to model congenital heart defects induced by pregestational diabetes. In response to high levels of glucose, organoids recapitulated the pathological arrhythmic phenotype, the macrosomia, and metabolic dysregulation (e.g., reduction in mitochondria, reduction in oxygen consumption, and increased lipid deposition). The same organoid model allowed for in vitro recapitulation of the hallmarks of pregestational diabetes [54].

More recently, Wang et al. [55] proposed an organoid model to study the effects of viral cardiomyopathy, caused by SARS-CoV-2. The demonstrated effects of the in vitro infection on modulating the transcriptomic signature, highlighting the effects on the pathways linked to cell adhesion, calcium handling, and cardiac hypertrophy, open the door to further applications of this model.

Finally, we discuss here briefly some applications of advanced in vitro models to recapitulate the effect of chemical and mechanical stimuli on cardiac tissue remodeling, often acting as a co-player in primary and secondary cardiomyopathies.

Lee et al. [56] stimulated MT composed of CMs and mesenchymal stem cell-derived fibroblasts by long-term (2 weeks) administration of TGF-β1. As expected, they observed the activation of myofibroblasts, with the overexpression of α-smooth muscle actin (α-SMA) and enhanced collagen deposition, with the subsequent arrhythmic phenotype, increased apoptosis of CM, and disruption of the mitochondrial network.

The integration of chemical and mechanical stimuli has been mainly achieved through lab-on-chip approaches, but this has still not reached the relevant three dimensionality of the constructs, in which several cell types are co-cultured without reproposing the complexity of the native cardiac tissue. Nevertheless, these approaches have the advantage of integrating complex stimuli, allowing for the read out of different parameters, using standardized protocols. For example, in the work of Mastikhina et al. [57], the authors monitored the on-chip co-culture of CFs and CMs derived from iPSCs by measuring on-line tissue contractile functionality. Moreover, they observed relevant fibrosis hallmarks, including increased tissue stiffness, induced brain natriuretic peptide secretion, enhanced collagen deposition, and the loss of contractile function. Despite the relevance and the significance of these approaches, they represent an abstraction of the complexity of the cardiac tissue and of the heart as an organ. Indeed, is clear that the simulation of hearth loads by unidirectional stretch or contraction is a simplification. In this regard, some attempts to design more complex organoids have been published, recapitulating the relevant cardiac geometrical features, such as the presence of cardiac chambers. Thanks to the design of a cardiac organoid model with one internal chamber, Ho et al. [58] achieved the in vitro recapitulation of cardiac hypertrophy. Indeed, the MTs responded to stimulation with endothelin-1, a biochemical stimulus known to induce hypertrophy through oxidative stress. Coherently with the in vivo scenario, MTs exhibited thickened chamber walls, reduced fractional shortening, and disarray of myofilaments and sarcomere proteins, thus inducing modifications in the calcium transient and, finally, tachy-arrhythmic phenotype.

**Table 2:** Summary of main studies proposing advanced in vitro modeling of non-genetic cardiomyopathies.

Non-Genetic Pathology	Technique	Cell Subsets	Ref.
MI	MT	iPSC-CM FB EC SC	[49]
MI	MT	iPSC-CM CF EC	[51]
MI	EHT	ESC-CM	[52]
Metabolic Disorders	CO	iPSC	[53]
Metabolic Disorders	CO	iPSC	[54]
Infection	CO	iPSC	[55]
Fibrosis	MT	ESC-CM ESC-MSC	[56]
Fibrosis	Microdevice	iPSC-CM iPSC-CF	[57]
Hypertrophy	MT	ESC-CM	[58]

## 4. In Vitro Modeling Cardiotoxicity

Advanced models have been widely proposed as test benches of cardiotoxicity, a highly increasing field of interest (Table 3). Cardiotoxicity can either result from exposition to toxic agents or being secondary to treatment with pharmaceutical compounds, such as chemotherapy drugs.

Doxorubicin is a common anti-cancer drug, with known cardiotoxic effects; thus, the reliability of novel designed in vitro models as in vitro platforms for drug screening is often assessed by testing their response to the administration of increasing doxorubicin doses. Examples are Polonchuk et al. [59] and Ergir et al. [60], who reported a coherent dose response to doxorubicin of their MT models. Kofron et al. [61] validated the potential of an MT method, composed of iPSC-CMs and CFs, for novel drug testing by assessing the proarrhythmic effect of eight drugs. All the tested drugs act as potassium channel (I_Kr_) blockers but with a different arrhythmic risk level, ranging from high to low, that was correctly stratified in vitro by the proposed model through the quantification of the MT action potential waveform-derived parameters. The additional effect of acute exposure to bisphenol A, a known toxic environmental pollutant, was also recapitulated. The achievement of these results was possible because MT recapitulated channels relevant to CMS signaling.

Tian et al. [62] reported the use of an iPSC organoid model to recapitulate the complex interplay between different pathways in regulating cardiac fibrosis. The work offers an example of the potential of in vitro models in predicting the combined effect of multiple treatments and potential cardiotoxicity.

In this respect, the work from Visone et al. [63] described the implementation of an MT-based lab-on-chip approach for drug screening. In particular, they designed an International Council for Harmonisation of Technical Requirements for Pharmaceuticals for Human Use guideline-compliant pharmacological campaign to monitor both cardiotoxic and proarrhythmic effects of 11 compounds acting on ion channels. By monitoring MT electrophysiological parameters, they could detect the arrhythmic events, observing a dose/compound-dependent response.

Significant interest has arisen recently for the study of the biological effects of the widespread environmental diffusion of microplastics. The polystyrene microplastic-induced hypertrophy in cardiac organoids obtained from an embryonic stem cell line is the focus of the study by Zhou et al. [64]: exposure to microplastics induced the upregulation of hypertrophy-related genes (e.g., *MYH*7*B*), the presence of edema, an increase in organoid size, apoptosis, and an inflammatory response.

By means of MTs, composed of iPSC-CMs, CFs, and ECs, Ma et al. [65] investigated the proarrhythmic effect of bisphenol A, a reagent used in the production of several plastic products. In particular, they could reproduce the high reactivity to this reagent of patients affected by long QT syndrome (resulting in arrhythmic phenotype) in comparison to healthy subjects. The same team in a second study [66] used the same model to investigate the pro-hypertrophic effect of bisphenol A, verifying its synergistic effects with treatment by endothelin-1 on Ca^2+^ transients and contractility.

## 5. Current Limitations and Future Perspectives

The exploitation of the high potential of iPSC technology for the efficient in vitro recapitulation of relevant pathophysiological scenarios still depends on some of the technological aspects.

The design of integrated in vitro models able to simulate the natural human cardiac environment, to recreate the progression of the pathology, and to evaluate the efficacy of possible treatments is clearly a required, but still not fully achieved, step forward in the field of cardiac in vitro modeling. However, this requires deep deliberation regarding the definition of the right balance between system simplicity and model thoroughness.

Several of the results described in this review are obtained by means of MTs or EHTs. Both methods require 2D on-plastic differentiation of iPSCs in different cardiac cell subsets or a culture of primary cells, making protocols long, costly, and labor-intensive. The results shown indicate that the level of iPSC-derived cell maturity in the used 3D systems is increased, and it could be sufficient to recapitulate some relevant features thanks to the presence of functional ion channels, organized sarcomeres, or more active physiological metabolic processes [67,68,69,70,71]. Nevertheless, significant cardiac features are still often underrepresented in these models. Only a few protocols recapitulate the myocardium geometrical structure (e.g., often MTs are aggregates of cell subsets, without specific cell alignment or the random alteration of cell types) and integrate relevant myocardium support systems, such as vasculature, innervation, the immune system, or, at least, inflammatory cells. Moreover, only rare examples reproduce the complexity of the heart as an organ, including features such as the presence of an endocardium or epicardium layer or cardiac chambers. In addition, they often find applicability in recapitulating cardiogenesis or early stages of congenital heart diseases due to their limited maturity level.

Several of these limitations could be overcome through the integration of engineered-scaffold-based techniques: bioprinting or electrospun deposition methods already showed their potential for the generation of substrates reproducing nanotopographic (e.g., alignment) or mechanical (e.g., stiffness) features. Regarding the state of the art, despite the significant amount of literature describing these techniques, there are only a few examples of their implementation for the recapitulation of the cardiomyopathies that are the subject of this review. For example, the work of Macadangdang et al. [72] underlined the relevance of CM alignment for the recapitulation of the hypertrophic response in DMD-CM, and Ma et al. [73] measured the effect on the contractility of a causative mutation for HCM, but neither one or the other implemented their methods in a 3D scenario.

Another main issue to be discussed is protocol reproducibility and standardization: the field, mainly because it is at its initial developmental stages, is characterized by an absence of guidelines and standards. Different teams propose different 3D models, obtained following different protocols, thus making the comparison between results from different studies complex. The lack of standardization in the field becomes evident by reading the literature and observing the variety of meanings associated to words, such as “organoid”, “microtissue”, or “spheroid”. The list of critical factors starts with the variety of iPSC differentiation protocols in cardiac cell subsets adopted by different teams, which leads to the generation of cells with different maturity levels, thus generating a bias in their functionality and ability to integrate with other cells. Moreover, even within the same team, some protocols show high intrinsic batch variability, calling for a high number of replicates to validate the obtained results. Additionally, the used cell subsets and their proportions are often different between MTs or EHTs proposed by different teams, as evident also in the tables published here. This complicates both the comparison of the results obtained by different teams and the deduction of the results that are translatable to the clinical setting. The world of organoids is even more complex: in principle, by bypassing 2D iPSC differentiation, the protocols should gain reproducibility; but, regarding the state of the art, there is a variety of protocols proposed to guide the complexity of iPSC self-differentiation and self-organization in complex 3D structures. Deep characterization of these structures and evaluation of the potential and limits for modeling physio/pathological scenarios will be needed. The study by Visone et al. [63], who took care to design a study compliant with a standard, is interesting, because, beyond the proposed platform or the tested compounds, it draws the path for the exploitation of in vitro obtained results to the world of the pharmaceutical industry.

The concept of standardization is tightly linked to the possibility of monitoring a process, enabling decisions to be made based on collected data. Therefore, the in vitro system strength is enhanced by their capacity to integrate live monitoring and control, allowing for a non-destructive evaluation of cell functionality and maturation. Some of the more commonly used on-line methods monitor cell activity through metabolites (e.g., oxygen consumption), energy, and generated forces (e.g., motion vector analysis) [74]. EHT success is mainly liked to the possibility of acquiring, through relatively easy, non-destructive, video-based monitoring, relevant parameters, including frequency and force of contraction. On the other hand, MTs can be produced more easily, using less material, without requiring specific tools (e.g., PDMS posts) and can be monitored through live staining. A notable approach was described by Ma et al. [73]: using two-photon polymerization, they produced a filamentous matrix supporting the culture of iPSC-CM carrying an HCM causative mutation. The matrix, on one side, allowed for the modulation of substrate rigidity, thus mimicking the physio/pathological surrounding, and, on the other side, allowed for the non-destructive measurement of cell contraction forces. The implementation of similar techniques could lead toward the definition of more standardized and high-throughput 3D advanced modeling.

Standardization and monitoring would also serve the cause of the design of multiorgan platforms, modeling the complex integration between different tissues, mimicking the dynamic crosstalk, and leading toward a holistic in vitro approach.

In conclusion, taking advantage of innovative methods and technologies proposed daily, the exploitation of patient-derived iPSC-based models could finally fulfill its potential, from recapitulating the mechanisms acting during disease progression to in vitro evaluation of novel pharmaceutical compound toxicity and efficacy, finally leading iPSC-based personalized medicine to become a standard of care.

## Figures and Tables

**Table 3:** Summary of main studies proposing advanced in vitro modeling to study cardiotoxicity.

Cardiotoxicity	Technique	Cell Subsets	Ref.
Drug-induced	MT	iPSC-CM iPSC-CF EC	[59]
Drug-induced	MT	iPSC-CM	[60]
Drug-induced	MT	iPSC-CM CF	[61]
Drug-induced	CO	iPSC	[62]
Drug-induced	Micodevice	iPSC-CM CF	[63]
Enviroment-induced	CO	ESC	[64]
Enviroment-induced	MT	iPSC-CM CF EC	[65]
Enviroment-induced	MT	iPSC-CM CF EC	[66]

## Abbreviations

**Abbreviation**	**Meaning**
iPSC	Induced Pluripotent Stem Cell
CM	Cardiomyocyte
iPSC-	iPSC-Derived
ESC-	Embryonic Stem Cell-Derived
CF	Cardiac Fibroblast
EC	Endothelial Cell
SC	Stromal Cells
MT	Microtissue
EHT	Engineered Heart Tissue
CO	Cardiac Organoid
